# The levels of plasma low density lipoprotein are independent of cholesterol ester transfer protein in fish-oil fed F1B hamsters

**DOI:** 10.1186/1743-7075-2-8

**Published:** 2005-03-11

**Authors:** Pujitha P de Silva, Alka Agarwal-Mawal, Phillip J Davis, Sukhinder Kaur Cheema

**Affiliations:** 1 Department of Biochemistry, Memorial University of Newfoundland, St. John's, NL, A1B 3X9 Canada

## Abstract

**Background:**

Cholesterol ester transfer protein (CETP) plays a major role in regulating the levels of LDL- and HDL-cholesterol. We previously observed a fish-oil-induced elevation of low-density lipoprotein (LDL)-and very-low-density lipoprotein (VLDL)-cholesterol concentrations and a decrease in high-density lipoprotein (HDL)-cholesterol concentration in F1B hamsters. The molecular mechanism/s by which fish oil induces hyperlipidaemic effect was investigated in this study. We examined whether the effects of dietary fish oil on plasma lipoprotein concentrations are due to fish-oil-induced alterations in plasma CETP activity. MIX diet, a diet supplemented with a mixture of lard and safflower oil, was used as the control diet.

**Results:**

We found that fish oil feeding in hamsters reduced CETP mass as well as CETP activity. Increasing the dietary fat level of fish-oil from 5% to 20% (w/w) led to a further decrease in CETP mass. Supplementation with dietary cholesterol increased both CETP mass and CETP activity in fish-oil and MIX-diet fed hamsters. However, there was no correlation between CETP mass as well as CETP activity and LDL-cholesterol concentrations.

**Conclusion:**

These findings suggest that cholesterol ester transfer between HDL and LDL is not likely to play a major role in determining fish-oil-induced changes in LDL- and HDL-cholesterol concentrations in F1B hamsters. A possible role of reduced clearance of LDL-particles as well as dietary fat level and dietary cholesterol dependent changes in LDL-lipid composition have been discussed.

## Background

Fish oil, rich in n-3 polyunsaturated fatty acids (PUFA), is considered beneficial in lowering the risk of coronary heart disease [1,2]. The beneficial effects of n-3 PUFA are mainly due to reduction in plasma triacylglycerol and very-low-density lipoprotein (VLDL) levels [3,4]. However, the effect of fish oil on low-density lipoprotein (LDL)-cholesterol concentration is inconsistent [5]. High levels of LDL are strong predictors of coronary heart disease. Normolipidaemic subjects show reduction in plasma LDL-cholesterol concentrations following intake of fish oil diet [6,7], however, fish oil supplementation to hyperlipidaemic subjects causes an increase in LDL-cholesterol concentrations [8,9]. The fish-oil-induced increase in LDL-cholesterol concentration is also observed in animal studies [10].

One of the factors that determine LDL concentrations is reverse cholesterol transport pathway, which removes cholesterol from peripheral tissues and returns to the liver. Cholesterol ester transfer protein (CETP) plays a major role in this pathway to transfer cholesterol esters from high-density lipoproteins (HDL) to VLDL and LDL, in exchange for triacylglycerols [11,12]. Thus, increased CETP activity can cause elevation of LDL-cholesterol concentration while decreasing the HDL-cholesterol concentrations. A positive correlation between plasma CETP concentration and LDL-cholesterol concentration has been observed in primates [11-13]. Thus, fish-oil-induced elevation of LDL-cholesterol concentration might be explained by changes in plasma CETP concentration.

CETP mediated exchange of cholesterol esters for triacylglycerols occurs mainly through an equimolar heteroexchange mechanism, which leads to alterations in cholesterol ester: triacylglycerol ratio. In addition to plasma CETP concentration, fatty acid composition of lipoproteins also modifies reverse cholesterol transfer. Cholesterol esters with n-3 polyunsaturated fatty acyl groups are more likely to transfer compared to cholesterol esters with saturated and monounsaturated fatty acyl groups [14]. This effect might be attributed to change in transition temperature of the lipid core of lipoproteins with change in lipid composition [15].

We previously observed fish-oil-induced elevation of VLDL-and LDL-cholesterol concentrations and decrease in HDL-cholesterol concentration in F1B hamsters [16]. In this study, we examined whether the effects of dietary fish oil on plasma lipoprotein concentrations are due to fish-oil-induced alterations in plasma cholesterol ester transfer protein activity.

## Results

### LDL-lipid composition

The LDL-cholesterol, free cholesterol, cholesterol ester, phospholipid and triacylglycerol concentrations were measured in Bio F1B hamsters treated with fish-oil or MIX diet (Table 1). Fish-oil diet led to significantly higher concentrations of total cholesterol, cholesterol ester, free cholesterol, triacylglycerol and phospholipids compared to MIX diet. Increasing the dietary fat levels of fish oil from 5% w/w (low fat) to 20 % w/w (high fat) caused a significant increase in free cholesterol and LDL-triacylglycerol concentrations. On the contrary, LDL-cholesterol ester concentrations decreased significantly (p < 0.0001) when the amount of fat was increased in fish oil diet. There was no effect of increase in dietary fat levels on LDL-cholesterol, cholesterol ester, triacylglycerol or phospholipids concentrations in the hamsters fed with MIX diet. Cholesterol supplementation of the diet led to an increase in LDL-cholesterol, cholesterol ester, triacylglycerol and phospholipids concentrations in hamsters fed with fish-oil diet. However, cholesterol supplementation to the MIX diet had no effect on these parameters. Furthermore, the effect of dietary cholesterol was greater in low fat fish-oil diet fed hamsters compared to the high fat fish oil fed hamsters (Table 1).

**Table 1:** LDL-lipid composition of hamsters fed with various diets.

**LDL-Lipids**	**Diet**	**Chol**	**Low Fat 5%**	**High Fat 20%**	**Statistical significance** **(3 way ANOVA)**	
					
					**Factor**	***P *value**
Total Cholesterol (mmol/l)	FO	-	4.84 ± 0.61	4.57 ± 0.36	DT	0.0001
		+	8.84 ± 2.06	5.10 ± 1.00	DT × DL	0.0020
	MIX	-	1.54 ± 0.32	1.41 ± 0.25	DT × CHOL	0.0007
		+	1.27 ± 0.25	1.63 ± 0.48	DL × CHOL	0.0200
Free Cholesterol (mmol/l)	FO	-	0.45 ± 0.05	1.53 ± 0.46	DL	0.0001
		+	0.69 ± 0.17	1.99 ± 0.45		
	MIX	-	0.28 ± 0.10	0.70 ± 0.13		
		+	0.38 ± 0.14	0.58 ± 0.15		
Cholesterol Ester (mmol/l)	FO	-	4.08 ± 0.43	2.62 ± 0.84	DT	0.0001
		+	8.05 ± 1.96	3.07 ± 1.00	CHOL	0.0001
	MIX	-	0.87 ± 0.30	0.88 ± 0.29	DT × DL	0.0300
		+	0.80 ± 0.17	0.79 ± 0.17	DL × CHOL	0.0004
Triacylglycerol (mmol/l)	FO	-	0.65 ± 0.08	1.62 ± 0.27	DT	0.0001
		+	1.01 ± 0.34	1.53 ± 0.22	DT × DL	0.0001
	MIX	-	0.24 ± 0.03	0.22 ± 0.04	DL × CHOL	0.0200
		+	0.29 ± 0.07	0.17 ± 0.01		
Phospholipids (mmol/l)	FO	-	0.69 ± 0.10	0.66 ± 0.11	DT	0.0001
		+	0.98 ± 0.18	0.73 ± 0.10	CHOL	0.03
	MIX	-	0.37 ± 0.07	0.36 ± 0.06	DT × DL	0.03
		+	0.33 ± 0.05	0.36 ± 0.04	DT × CHOL	0.009

FO, fish oil; MIX, MIX diet; Chol, cholesterol; DT, diet type; DL, diet level; CHOL, Cholesterol For details of diets and procedures, see "Materials and methods" section. Animals were fed the indicated diets for 2 weeks. All values represent means ± SD.

**Figure 1:**
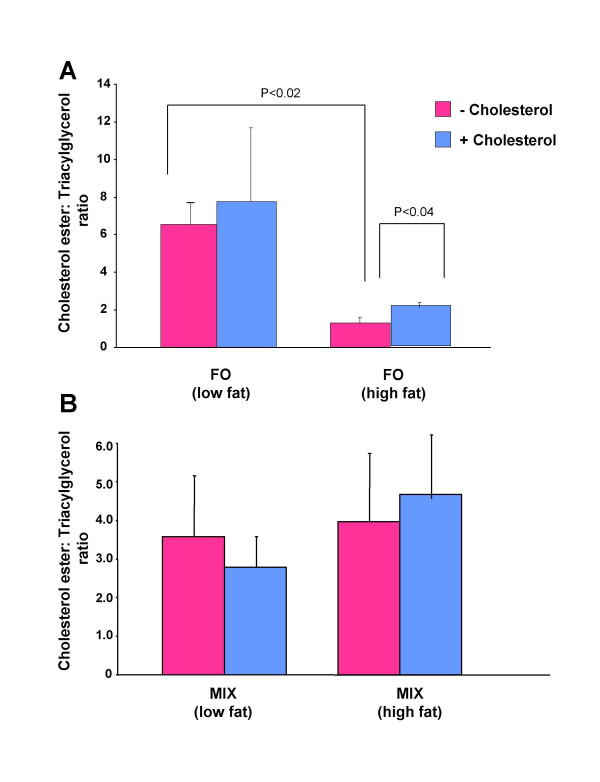
**Cholesterol ester: triacylglycerol ratio in fish-oil (A) and MIX-diet (B) fed hamsters**. Hamsters were fed fish oil (FO) or MIX diet at a low fat (5% w/w) or a high fat (20% w/w) level in the absence (pink) or presence (blue) of 0.25% w/w cholesterol. Lipids were analyzed as described in the methods. Values are means for 12 animals with standard deviations shown by vertical bars. Differences between groups were evaluated using Student's *t *test.

Change in dietary fat level altered the LDL-cholesterol ester: triacylglycerol ratio (Figure 1). There was a significant decrease in LDL-cholesterol ester: triacylglycerol ratio in fish-oil diet fed hamsters when the dietary fat level was increased from 5 % to 20 % (Figure 1A). However, there was no significant effect of dietary fat level on LDL-cholesterol ester: triacylglycerol ratio in the MIX diet fed hamsters (Figure 1B). Addition of cholesterol to the high fat fish-oil diet caused a slight but significant increase in LDL-cholesterol ester: triacylglycerol ratio compared to the high fat fish oil diet alone (Figure 1A). However, addition of cholesterol to the MIX diet had no significant effect on cholesterol ester: triacylglycerol ratio compared to hamsters fed the MIX diet alone (Figure 1B).

### HDL-lipid composition

Changes in HDL-lipid composition in Bio F1 B hamsters on different diets are given in Table 2. Fish-oil diet fed hamsters had significantly lower concentrations of HDL-cholesterol, free cholesterol, cholesterol ester and phospholipids compared to MIX-diet fed hamsters. However, there was no significant effect of fish-oil on HDL-triacylglycerol concentrations. Increasing the dietary fat levels from low fat to high fat caused a decrease in cholesterol ester concentrations in fish-oil diet fed hamsters. Cholesterol supplementation caused an increase in HDL-cholesterol and cholesterol ester concentrations irrespective of the diet type in low fat fed hamsters. However, there was no effect of dietary cholesterol on HDL-cholesterol concentration in high fat fed hamsters. Cholesterol supplementation led to a significant increase in phospholipids concentration for all type of diets, both at low and high fat levels.

**Table 2:** HDL-lipid composition of hamsters fed with various diets.

**HDL-Lipids**	**Diet**	**Chol**	**Low Fat 5%**	**High Fat 20%**	**Statistical significance (3 way ANOVA)**	
					
					**Factor**	***P *value**
Total Cholesterol (mmol/l)	FO	-	2.90 ± 0.29	2.19 ± 0.48	DT	0.0001
		+	3.96 ± 0.87	2.24 ± 0.56	CHOL	0.0020
	MIX	-	4.74 ± 0.07	5.86 ± 0.37	DT × DL	0.0001
		+	6.00 ± 1.23	6.78 ± 0.45		
Free Cholesterol (mmol/l)	FO	-	0.18 ± 0.08	0.31 ± 0.07	DT	0.0001
		+	0.31 ± 0.08	0.25 ± 0.16		
	MIX	-	0.60 ± 0.17	0.57 ± 0.03		
		+	0.60 ± 0.17	0.59 ± 0.12		
Cholesterol Ester (mmol/l)	FO	-	2.67 ± 0.29	1.88 ± 0.41	DT	0.0001
		+	3.88 ± 0.71	1.98 ± 0.49	CHOL	0.0009
	MIX	-	3.55 ± 0.61	4.34 ± 0.42	DT × DL	0.0001
		+	4.33 ± 0.80	5.34 ± 0.96		
Triacylglycerol (mmol/l)	FO	-	0.32 ± 0.03	0.44 ± 0.12	DT × DL	0.0400
		+	0.39 ± 0.14	0.33 ± 0.10	DL × CHOL 0.0400	
	MIX	-	0.37 ± 0.03	0.32 ± 0.03		
		+	0.40 ± 0.09	0.30 ± 0.04		
Phospholipids (mmol/l)	FO	-	0.57 ± 0.12	0.59 ± 0.16	DT	0.0001
		+	1.62 ± 0.43	1.27 ± 0.23	DL	0.01
	MIX	-	3.43 ± 0.26	2.91 ± 0.33	CHOL	0.0003
		+	4.20 ± 0.45	4.67 ± 0.46	DT × DL	0.0005
					DT × CHOL 0.05	
					DL × CHOL 0.0007	

FO, fish oil; Mix, MIX diet; Chol, cholesterol; DT, diet type; DL, diet level; CHOL, cholesterol. For details of diets and procedures, see "Materials and methods" section. Animals were fed the indicated diets for 2 weeks. All values represent means ± SD.

### Surface lipid to core lipid ratio for LDL and HDL

The LDL surface lipids (free cholesterol and phospholipids) to core lipids (cholesterol esters and triacylglycerols) ratio was higher in MIX diet fed hamsters compared to hamsters fed the fish oil diet, at both low and high fat levels (Table 3). Increasing the quantity of fat in the diet increased the surface to core lipid ratio, whereas addition of dietary cholesterol had no significant effect, in both fish oil and MIX diet fed hamsters (Table 3). The HDL surface lipid to core lipid ratio was also higher in MIX diet fed hamsters compared to the fish oil fed hamsters, at both low fat and high fat levels (Table 3). Addition of cholesterol to the fish oil diet caused a significant increase in surface lipid to core lipid ratio, at both low and high fat levels, whereas no significant effect of dietary cholesterol was observed for the MIX diet (Table 3).

**Table 3:** Surface lipid to Core lipid ratio of LDL and HDL particles from hamsters fed with various diets.

Surface lipid: Core lipid					
Particle Type	Fat level	Fish Oil		Mix	

		- Cholesterol	+ Cholesterol	- Cholesterol	+ Cholesterol
LDL	5%	0.24	0.18	0.59	0.65
	20%	0.52	0.59	0.96	0.98
HDL	5%	0.25	0.45	1.02	1.01
	20%	0.39	0.69	0.75	0.93

Surface lipids are composed of free cholesterol and phospholipids. Core lipids are composed of cholesterol esters and triacylglycerols. The surface lipid to core lipid ratio was calculated by dividing the mean surface lipids with mean core lipids.

### CETP activity and CETP mass

**Figure 2:**
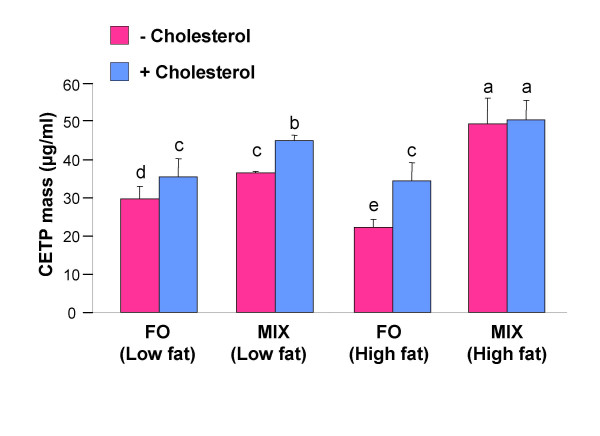
**Plasma CETP mass in fish-oil and MIX-diet fed hamsters**. Plasma CETP mass in hamsters fed with a fish-oil (FO) or a MIX-diet at a low fat (5% w/w) or a high fat (20% w/w) level in the absence (pink) or presence (blue) of 0.25% w/w cholesterol. Plasma was collected and assayed for CETP mass using ELISA as described in the methods. Values are means for 12 animals with standard deviations shown by vertical bars. Differences between groups were evaluated using 3-way ANOVA. Values without a common superscript are significantly different from each other.

**Figure 3:**
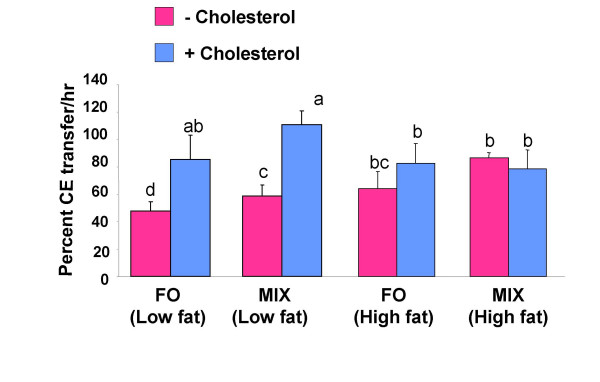
**Plasma CETP activity in fish-oil and MIX-diet fed hamsters**. Plasma CETP activity in hamsters fed with a fish-oil (FO) or a MIX-diet at a low fat (5% w/w) or a high fat (20% w/w) level in the absence (pink) or presence (blue) of 0.25% w/w cholesterol. Plasma was collected and assayed for CETP activity using a radioisotope method as described in the methods. Values are means for 12 animals with standard deviations shown by vertical bars. Differences between groups were evaluated using 3-way ANOVA. Values without a common superscript are significantly different from each other.

It has been shown previously that there is a correlation between the level of CETP and that of LDL-cholesterol. To further investigate our observation of fish-oil-induced elevation of LDL-cholesterol concentrations and a decrease in HDL-cholesterol concentration in F1B hamsters, we measured CETP mass as well as CETP activity. The changes in CETP mass and CETP activity are shown in Figure 2 and 3 respectively. Treatment of hamsters with fish-oil diet led to a decrease in CETP mass (p < 0.0001) and CETP activity (p < 0.002) compared to MIX diet, at low fat level. Increasing the dietary fat levels from low fat to high fat caused a further decrease in CETP mass in fish-oil diet fed hamsters, whereas same change brought an increase in CETP mass in MIX diet fed hamsters (DT × DL interaction, p < 0.002). On the other hand, plasma CETP activity increased in both fish-oil and MIX-diet fed hamsters by increasing the dietary fat levels. Dietary cholesterol caused an increase in CETP mass (p < 0.0002) and CETP activity (p < 0.0001) in both fish-oil and MIX-diet fed hamsters at low fat level. However, cholesterol mediated increase in CETP activity was not observed at high fat levels for both fish oil and MIX diets.

### Correlation between CETP mass and CETP activity

**Figure 4:**
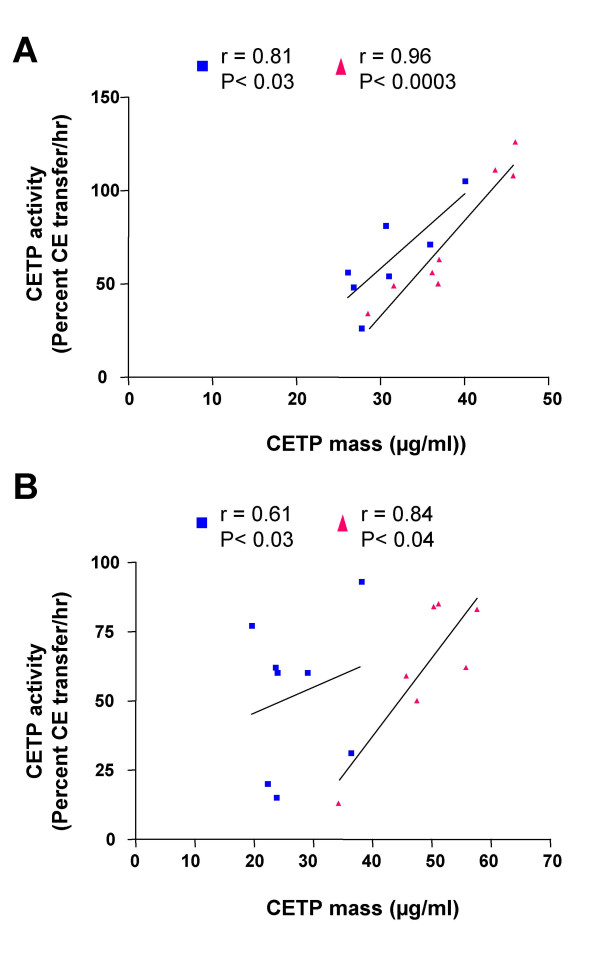
**Correlation between CETP activity and CETP mass in fish-oil and MIX-diet fed hamsters**. Correlation between CETP activity and CETP mass in hamsters fed with a fish-oil (blue) or a MIX (red) diet, supplemented with low fat (5% w/w) (panel A) or high fat (20% w/w) (panel B). Plasma was collected and assayed for CETP activity and CETP mass as described in the methods.

CETP mass was significantly and positively correlated with CETP activity in low fat fish oil (r = 0.81, p < 0.03) and MIX diet (r = 0.96 p < 0.0003) fed hamsters (Figure 4A) and high fat MIX diet fed hamsters (r = 0.84, p < 0.02) (Figure 4B). The correlation between CETP mass and CETP activity in high fat fish-oil fed hamsters was not significant. This indicates that CETP activity is an accurate reflection of CETP mass in both fish-oil and MIX-diet fed hamsters at low fat levels.

### Correlation between CETP and LDL-lipid levels

**Figure 5:**
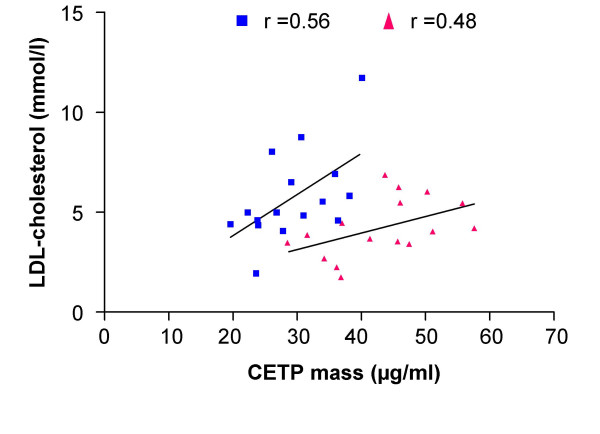
**Correlation between CETP mass and LDL-cholesterol in fish-oil and MIX-diet fed hamsters**. Correlation between CETP mass and LDL-cholesterol in hamsters fed with a fish-oil (blue) or a MIX (red) diet. Plasma CETP mass and LDL-cholesterol concentration were assayed as described in the methods.

**Figure 6:**
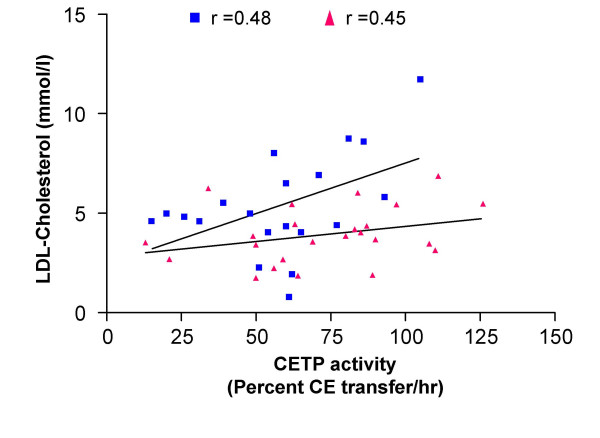
**Correlation between CETP activity and LDL-cholesterol in fish-oil and MIX-diet fed hamsters**. Correlation between CETP activity and LDL-cholesterol in hamsters fed with a fish-oil (blue) or a MIX (red) diet. Plasma CETP activity and LDL-cholesterol concentration were assayed as described in the methods.

The correlations between CETP mass, CETP activity and LDL-cholesterol are shown in Figure 5 and 6 respectively. There was no significant correlation between CETP mass and LDL-cholesterol concentrations in fish-oil diet (r = 0.56) or MIX diet (r = 0.48) fed hamsters. Similarly, the CETP activity was not significantly correlated with LDL-cholesterol concentrations in fish-oil (r = 0.48) or MIX diet (r = 0.45) fed hamsters.

## Discussion

CETP plays an important role in maintaining the levels of plasma LDL and HDL [12]. Increased levels of plasma CETP cause an increase in the transfer of cholesterol esters from HDL to LDL, thereby raising the levels of LDL. In recent years, antibodies have been raised against CETP as a therapy to lower plasma LDL-levels and to prevent cardiovascular disease [12]. Fish oil has also gained much interest in the recent years as a therapy against cardiovascular disease. Earlier, we observed that fish oil caused an increase in plasma lipid and lipoprotein levels in a unique animal model, the F1B hamster [16]. In this study, we investigated whether the increase in plasma LDL-levels in fish-oil fed F1B hamsters is due to an increase in plasma CETP activity. Fish oil caused an increase in LDL-cholesterol concentration and a decrease in HDL-cholesterol concentration compared to hamsters fed a MIX diet (Table 1 and 2). However, CETP activity and CETP mass were lower in fish-oil fed hamsters compared to the MIX-diet fed hamsters. Earlier studies in human subjects also show fish-oil-induced reduction in CETP activity compared to safflower oil fed counter parts [17,18]. Moreover, we did not find significant correlation between CETP mass as well as CETP activity and LDL-cholesterol in this study. Data presented here suggests that cholesterol ester transfer between HDL and LDL is not likely to play a major role in determining fish-oil-induced changes in LDL- and HDL-cholesterol concentrations.

Cholesterol esters are more liable to transfer in fish-oil fed subjects since the core of the lipoprotein particles from these subjects have lower transition temperature [15] due to abundance of long chain polyunsaturated fatty acids. This theory is in agreement with the finding that there is increased cholesterol ester mass transfer in eicosapentaenoic acid (omega-3 20:5, EPA) fed rabbits while there was no change in plasma CETP activity [14]. We did not measure cholesterol ester mass transfer to determine the contribution of HDL-and LDL-fatty acid composition in order to explain fish-oil- induced elevation of LDL-cholesterol concentration. However, our data shows that the LDL and HDL surface lipid to core lipid ratio is significantly lower in fish oil fed hamsters than the MIX diet fed hamsters, at both low and high fat levels. Thus, the composition of the LDL and HDL particles appears to be significantly different between the fish oil and MIX diet fed hamsters. It has previously been shown that the LDL particle composition is an important determinant of LDL clearance [19]. In our previous study [16] we have shown that hepatic LDL-receptor mRNA levels were significantly low in fish-oil fed hamsters. In addition there are reports that in cases of CETP deficient subjects, LDL-particles have reduced affinity for LDL-receptor [20]. One can hypothesize that the increase in both LDL-cholesterol and triacylglycerol concentrations (Table 1) in fish-oil fed hamsters may points towards suppression of LDL clearance rather than increased cholesterol ester/triacylglycerol exchange as the cause.

Increasing dietary fat level of fish oil decreased cholesterol ester transfer as reflected by decrease in CETP mass (Figure 2). Thus, dietary fat level dependent reduction in LDL-cholesterol ester: triacylglycerol ratio in fish-oil diet fed hamsters (Figure 1A) might be due to the decrease in cholesterol ester transfer. However, in contrast to CETP mass, CETP activity slightly increased with the increase of dietary fat level. The plasma used for CETP activity assay in high fat fish-oil fed hamsters contained very high levels of chylomicrons, VLDL and LDL, which are potential acceptors of cholesterol esters from radiolabeled exogenous HDL [21]. Thus, CETP activity of high fat fish-oil fed hamsters might be an exaggerated reflection of CETP mass.

HDL-total cholesterol and cholesterol ester concentrations were significantly lower in fish-oil diet fed hamsters compared to MIX-diet fed hamsters, while there was no difference in HDL-triacylglycerol concentrations (Table 2). Previous studies using hamsters [22,23] and non-human primates [24] have shown decreased HDL-cholesterol concentrations following fish oil feeding, which support our observation. Plasma HDL-cholesterol concentration is mainly regulated by reverse cholesterol transport pathway and cellular cholesterol efflux [25,26]. The reduction of HDL-cholesterol concentration, while decrease in cholesterol ester transfer in fish-oil fed hamsters compared to MIX-diet fed hamsters implicates that fish-oil-induced HDL-cholesterol lowering effect is not due to the changes in cholesterol ester transfer, but might be attributed to decreased efflux of cholesterol from peripheral cells.

Increasing the dietary fat level from low fat to high fat caused a decrease in HDL-total cholesterol and cholesterol ester levels (Table 2), which might be due to poor esterification of HDL-free cholesterol-to-cholesterol esters by lecithin-cholesterol-acyl-transferase (LCAT) as n-3 PUFA are known to be less utilized by LCAT for the formation of cholesterol esters [27,28]. Dietary cholesterol supplementation led to an increase in HDL-cholesterol concentration and also caused an increase in CETP mass/activity in both fish-oil and MIX-diet fed hamsters. This finding suggest that cholesterol mediated increase in HDL-cholesterol was not associated with changes in cholesterol ester transfer, but might be due to other factors, possibly due to an increased cholesterol efflux from peripheral cells.

The interactive effect of dietary cholesterol and n-3 PUFA on plasma cholesterol ester transfer is not yet known. Cholesterol supplementation caused an increase in plasma CETP activity in both fish-oil and MIX-diet fed hamsters at low fat levels (Figure 3), which is consistent with other reports [29]. These observations suggest that the regulation of CETP is dependent on the presence of dietary cholesterol. Dietary cholesterol is known to increase plasma CETP concentrations [29]. The increase in plasma CETP concentrations in response to dietary cholesterol is due to an increase in CETP mRNA levels in adipose tissue and liver [30,31]. In hamsters, CETP is mainly expressed in the adipose tissue, and the cholesterol mediated increase in plasma CETP activity is directly related to an increase in adipose tissue CETP mRNA levels [31]. Our findings are consistent with the previous observations that the regulation of CETP is dependent on dietary cholesterol. However, our observations show that the supplementation of cholesterol to the high fat fish oil and MIX diet had no significant effect on CETP activity as compared to high fat fish oil and MIX diet alone (Figure 3). These findings suggest that high fat diets interfere with cholesterol to regulate CETP, which is similar to the observations made for the regulation of the human CETP gene (under publication).

## Conclusion

In summary, fish oil induced increase in LDL-cholesterol concentration in F1B hamsters, as well as effects of diet type, diet fat level and dietary cholesterol level on HDL-lipids were not associated with changes in plasma cholesterol ester transfer activity. It is likely that the dietary fat composition altered the LDL-core lipid composition, which in turn inhibited the uptake of LDL particles. This in combination with decrease of LDL-receptor mRNA levels may be the likely cause of increased plasma LDL-levels in fish-oil fed F1B hamsters.

## Methods

### Animals and diets

The F1B hamsters (7 weeks old) were obtained from Bio Breeders Inc. (Water Town, MA) and kept on chow diet for one week prior to feeding specific diets. After this equilibration period, hamsters were divided into 8 groups (n = 12) and each group was fed with one of the specified diets. The specified diets consisted of fat free semi-purified diet (ICN Biomedical Inc., OH) that was supplemented with either fish oil (menhaden oil, Sigma Chemical Co., St. Louis, MO) or a mixture of lard and safflower oil in 1.5:1 ratio (MIX diet) [16]. The fat content of the diets was either 5% w/w (low fat) or 20% w/w (high fat). Due to the presence of cholesterol in fish oil, the low fat fish oil diet contained 0.025% w/w of cholesterol, and the high fat fish-oil diet contained 0.1% w/w of cholesterol. Thus, the same amount of cholesterol was added to the low fat and the high fat MIX diets to keep cholesterol content similar. For the high cholesterol diets, the fish oil and MIX diets were supplemented with additional cholesterol to bring the final concentration of cholesterol to 0.25% w/w. All diets were stored at -20°C and animals were given fresh diets each day.

The animals were maintained on specific diets for 2 weeks ad libitum. The food intake was measured daily during the study period, and the body weight was checked at the beginning of the study period, one week later and at the conclusion of the study. There was no difference in food intake and body weight gain between different diet groups. All animals were housed in individual cages in a single room with enriched environment. The Institutional Animal Care use Committee (IACC) approved all experimental procedures, which are in accordance with the principles and guidelines of the Canadian Council on Animal Care. After two weeks on specified diets the animals were sacrificed after 14 hrs of fasting. Blood was collected by cardiac puncture into tubes containing EDTA and centrifuged immediately to separate plasma. Plasma samples were stored at 4°C on ice until further use.

### Lipoprotein separation and analysis

Plasma was centrifuged at 15,500 *g *for 20 min at 12°C [32] to separate chylomicrons. The infranatant was separated and used for isolation of other lipoproteins fractions i.e. VLDL, LDL and HDL by sequential density ultra centrifugation [33]. Isolated individual lipoprotein fractions i.e. VLDL, LDL and HDL were stored at 4°C for further analysis.

Total cholesterol concentration was assayed in all lipoprotein fractions using cholesterol assay kit # 402 (Sigma Diagnostics Inc, St. Louis, MO). Total triacylglycerol concentration of lipoprotein fractions was assayed using triglyceride assay kit # 344 (Sigma Diagnostics Inc, St. Louis, MO). Free cholesterol in plasma and individual lipoprotein fractions was assayed using free cholesterol assay kit (Wako Chemicals, VA). Cholesterol ester concentration was determined by subtracting the free cholesterol concentration from total cholesterol concentration. Phospholipids were analyzed using the method of Bartlett [34].

### Cholesterol ester transport protein (CETP) activity assay

CETP in the plasma samples was assayed by a radioactive method [21] that was modified from a previously published method [35]. LDL- and HDL-lipoprotein fractions were isolated from human plasma [36] and the HDL-fraction was radiolabeled, using ^14^C-cholesterol oleate [35]. Plasma (5 μl) was combined with radiolabeled HDL (5 μg), LDL (50 μg) and 115 μl of incubation buffer (10 mM Tris, 150 mM NaCl, 2 mM EDTA) and incubated at 37°C for 1 hr. The LDL fraction was separated by heparin-manganese precipitation. The radioactivity in the supernatant and the precipitate was counted and the results were expressed as percent cholesterol ester(CE) transferred/hour.

### CETP mass

CETP mass in plasma samples was quantitatively assayed using CETP ELISA- DAIICHI kit (Daiichi Pure Chemicals Co., LTD, Tokyo) as previously published [21]. Concentrations of the samples were calculated using a standard curve developed using a CETP stock solution with known concentration.

### Statistical analysis

The effect of diet type, dietary fat level and dietary cholesterol was determined using 3-way analysis of variance, and a Tukey's post hoc test was used to test significant differences revealed by the ANOVA. Values are group means ± SD, n = 12; Differences were considered to be statistically significant if the associated *P *value was <0.05 [37].

## Competing interests

The author(s) declare that they have no competing interests.

## Authors' contributions

PPD conducted all the experiments in this study. PPD and AAM analyzed and interpreted the data, as well as drafted the manuscript. PJD is a collaborator on this project. SKC is the Principal investigator, has conceived the study, participated in its design and final approval of the version to be published. All Authors read and approved the final manuscript.
